# Small lesion depiction and quantification accuracy of oncological ^18^F-FDG PET/CT with small voxel and Bayesian penalized likelihood reconstruction

**DOI:** 10.1186/s40658-022-00451-5

**Published:** 2022-03-26

**Authors:** Lei Xu, Ru-Shuai Li, Run-Ze Wu, Rui Yang, Qin-Qin You, Xiao-Chen Yao, Hui-Fang Xie, Yang Lv, Yun Dong, Feng Wang, Qing-Le Meng

**Affiliations:** 1 Department of Nuclear Medicine, Nanjing First Hospital, Nanjing Medical University, 68 Changle Road, Nanjing, 210006 China; 2 United Imaging Healthcare, 2258 Chengbei Road, Shanghai, 201870 China

**Keywords:** FDG, PET, Lung nodule, Small voxel reconstruction, Bayesian penalized likelihood reconstruction, Small lesion detection

## Abstract

**Background:**

To investigate the influence of small voxel Bayesian penalized likelihood (SVB) reconstruction on small lesion detection compared to ordered subset expectation maximization (OSEM) reconstruction using a clinical trials network (CTN) chest phantom and the patients with ^18^F-FDG-avid small lung tumors, and determine the optimal penalty factor for the lesion depiction and quantification.

**Methods:**

The CTN phantom was filled with ^18^F solution with a sphere-to-background ratio of 3.81:1. Twenty-four patients with ^18^F-FDG-avid lung lesions (diameter < 2 cm) were enrolled. Six groups of PET images were reconstructed: routine voxel OSEM (RVOSEM), small voxel OSEM (SVOSEM), and SVB reconstructions with four penalty factors: 0.6, 0.8, 0.9, and 1.0 (SVB0.6, SVB0.8, SVB0.9, and SVB1.0). The routine and small voxel sizes are 4 × 4 × 4 and 2 × 2 × 2 mm^3^. The recovery coefficient (RC) was calculated by dividing the measured activity by the injected activity of the hot spheres in the phantom study. The SUV_max_, target-to-liver ratio (TLR), contrast-to-noise ratio (CNR), the volume of the lesions, and the image noise of the liver were measured and calculated in the patient study. Visual image quality of the patient image was scored by two radiologists using a 5-point scale.

**Results:**

In the phantom study, SVB0.6, SVB0.8, and SVB0.9 achieved higher RCs than SVOSEM. The RC was higher in SVOSEM than RVOSEM and SVB1.0. In the patient study, the SUV_max_, TLR, and visual image quality scores of SVB0.6 to SVB0.9 were higher than those of RVOSEM, while the image noise of SVB0.8 to SVB1.0 was equivalent to or lower than that of RVOSEM. All SVB groups had higher CNRs than RVOSEM, but there was no difference between RVOSEM and SVOSEM. The lesion volumes derived from SVB0.6 to SVB0.9 were accurate, but over-estimated by RVOSEM, SVOSEM, and SVB1.0, using the CT measurement as the standard reference.

**Conclusions:**

The SVB reconstruction improved lesion contrast, TLR, CNR, and volumetric quantification accuracy for small lesions compared to RVOSEM reconstruction without image noise degradation or the need of longer emission time. A penalty factor of 0.8–0.9 was optimal for SVB reconstruction for the small tumor detection with ^18^F-FDG PET/CT.

**Supplementary Information:**

The online version contains supplementary material available at 10.1186/s40658-022-00451-5.

## Background

^18^F-FDG PET/CT is widely used in oncology imaging to measure metabolic activities [1]. However, PET’s ability to detect small tumors is limited by its spatial resolution and partial volume effects [2]. To alleviate this constraint, small voxel reconstruction with a voxel size of ≤ 2 × 2 × 2 mm^3^ was proposed for whole-body PET imaging [3, 4]. Small voxel reconstructions improved the detection of in-transit metastases in melanoma patients [5], the contrast and the localization of small spheres in the phantom [6], and the diagnostic performance of PET/CT in tumor patients [7]. However, the image noise was inherently high for small voxel reconstructions because of lower counts per voxel, requiring a longer emission time to compensate for the lower count statistics [5–7].

Bayesian penalized likelihood (BPL) reconstructions can reduce the image noise and increase the contrast recovery, allowing for more accurate lesion quantification compared to standard ordered subset expectation maximization (OSEM) reconstruction [8, 9]. BPL reconstruction improved spatial resolution [10] and lesion contrast for small lung nodules [11–13], and the detectability of sub-centimeter tumors [14, 15].

We hypothesize that combining small voxel and BPL (SVB) reconstruction will further improve the depiction and the quantification accuracy of small tumors without the image noise degradation or the need of a longer emission time. However, the performance of SVB reconstruction on small lesions has not been fully explored yet. Therefore, our study aimed to investigate whether SVB reconstruction can accurately depict small lung tumors with a diameter of less than 2 cm on ^18^F-FDG PET/CT. We compared quantitative and qualitative image quality metrics between SVB reconstruction with 2 × 2 × 2 mm^3^ voxel and OSEM reconstruction with 4 × 4 × 4 mm^3^ voxel using data from a chest phantom and the patients. The image quality metrics, including contrast recovery, volumetric measurement accuracy, and the lesion depiction capability, were evaluated to determine the optimal penalty factor for SVB reconstruction.

## Methods

### Phantom study

A clinical trials network (CTN) anthropological chest phantom of Society of Nuclear Medicine and Molecular Imaging was used which had two polystyrene-filled chambers and a uniform background to mimic the lung and the other soft tissues in the chest. The phantom background was filled with ^18^F solution with an activity concentration of 6.2 kBq/ml. Seven spheres (diameters = 7, 10, 13, 17, 22, 28, and 37 mm) were placed in the phantom background and five spheres (2 spheres of 10-mm diameter and 3 spheres of 13, 17, and 22-mm diameter) in the polystyrene-filled chambers. A figure was provided in the Additional file 1 to illustrate the location of the spheres in the phantom. All spheres were filled with ^18^F solution at a sphere-to-background ratio of 3.81:1.

### Patient study

This retrospective study included 29 patients undergoing ^18^F-FDG PET/CT examination for tumor staging or re-staging. The inclusion criteria were: the radiological report indicated there were ^18^F-FDG-avid lesions in the lungs, the target lesion could be segmented on CT images with a diameter of less than 2 cm, and the list-mode data were available for additional reconstructions. The exclusion criteria were: the respiratory motion significantly affected quantification accuracy of the lesion on PET images (n = 1); PET data were acquired at a late phase that had an uptake time longer than 90 min (n = 4). Finally, 24 patients were enrolled. The study was approved by the Institutional Ethics Committee, and the informed consent was waivered due to the retrospective nature of this study.

### PET/CT procedure for the phantom and patient study

All images were acquired with a digital PET/CT scanner (uMI780, United Imaging Healthcare, Shanghai, China) equipped with lutetium–yttrium orthosilicate crystal coupled with silicon (Si) photomultipliers (PM). The patients were asked to fast a least 6 h before the PET/CT examination. The patients received an injection of the ^18^F-FDG solution according to their body weight (5.0 MBq/kg). The PET/CT examination was started 60 min after the injection. The phantom and the patients underwent a CT scan using a tube voltage of 120 kVp and a tube current of 250 mAs with automatic modulation, followed by a whole-body PET scan with an emission time of 2 min per bed position from the skull base to the upper thigh of the patient or about 30 cm long to cover the whole phantom.

### PET/CT image reconstruction

The PET images were reconstructed into six groups: routine voxel OSEM (RVOSEM), small voxel OSEM (SVOSEM), and SVB reconstructions with four penalty factors: 0.6, 0.8, 0.9, and 1.0 that were referred to as SVB0.6, SVB0.8, SVB0.9, and SVB1.0, respectively. The RVOSEM group used the standard reconstruction protocol of our department with the following parameters: a field of view (FOV) of 512 mm, a reconstruction matrix of 128 × 128, a slice thickness of 4 mm (i.e., 4 × 4 × 4 mm^3^ voxel size), OSEM with 2 iterations, 20 subsets, and 3 mm Gaussian post-filtering. Furthermore, all SVB groups and the SVOSEM group were reconstructed by using a FOV of 512 mm, a reconstruction matrix of 256 × 256, and a slice thickness of 2 mm (i.e., 2 × 2 × 2 mm^3^ voxel size). The SVB groups used total variation regularized expectation maximization (TVREM, or commercially known as HYPER Iterative, United Imaging Healthcare) reconstruction. TVREM is an implementation of BPL algorithms that incorporates a total variation regularizer and the sensitivity profile of PET scanners into the penalization term [9, 16, 17]. The Additional file 1 Section I provides more details about TVREM. The penalty factor is a hyper-parameter regulating the image contrast and smoothness that is operator-adjusted between 0 and 1. A penalty factor of 0.6 was selected as the lower bound in this study because we found it had a higher image noise than RVOSEM but still satisfied the image quality control criteria in a preliminary patient study, which was confirmed in the full study afterwards (Additional file 1: Fig. 2). A penalty of 1.0 was chosen because it is the upper limit and gives the maximal reduction in the image noise. All PET image reconstructions included time-of-flight model, point spread function, and other necessary corrections, such as the attenuation correction and the scatter correction. The CT images were reconstructed using a FOV of 700 mm, a reconstruction matrix of 512 × 512, and a slice thickness of 3 mm with 1.5 mm increments.

### Quantitative evaluation of the phantom and patient images

All quantitative evaluations were performed by a senior 3D laboratory technician supervised by a nuclear radiologist on a commercial medical image workstation (uWI-MI, United Imaging Healthcare). On the phantom images, a spherical volume of interest (VOI) was carefully placed on the hot sphere with the aid of CT images and fusion views. The diameter of the VOI was kept the same as the nominal diameter of each hot sphere. The mean activity was measured at each VOI, and the recovery coefficient (RC) was calculated by dividing the measured activity by the injected activity of the hot sphere. Moreover, eight spherical VOIs with a fixed diameter of 1.5 cm were placed in the uniform regions in the phantom background. Six of them were in the mediastinum, and two were in the heart and shoulder regions. A figure was provided in the Additional file 1 to illustrate the location of those VOIs. The standard deviation (SD) was calculated from the pixel values in each VOI as a quantitative metric of the image noise.

For each patient, a region of interest (ROI) with a diameter of 3 cm was placed on a homogeneous area in the right lobe of the liver. Another ROI with 1.5 cm diameter was placed in the mediastinal blood pool (aorta arch). The mean and SD were calculated from the pixel values in the ROIs placed in the liver and the mediastinum, respectively. Similar to the phantom study, the SDs of the liver and the mediastinum were used as the measurement of the image noise. All VOIs and ROIs were first placed on the RVOSEM images and propagated to the other groups.

A small ^18^F-FDG-avid lung lesion with a diameter of ≤ 2 cm measured on CT images was selected for each patient by the radiologist. The lesion does not have necrosis or other conditions causing non-metabolic tissue in the lesion and can be segmented on CT and PET images with a semi-automatic segmentation tool (MI-Oncology, United Imaging Healthcare, Shanghai China). The lesion’s MTV was obtained with a threshold-based segmentation method on the PET images using 41% of SUV_max_ as the threshold [1]. The lesion was subsequently segmented on the CT images, and its resulting volume was served as the standard reference in the comparison of PET- and CT-derived volume. The lesion SUV_max_ was documented. The tumor-to-background ratio (TBR) was calculated by dividing SUV_max_ of the lesion by SUV_mean_ of the liver. The contrast-to-noise ratio (CNR) of the lesion was calculated by dividing the absolute difference of the lesion SUV_max_ and the mediastinum SUV_mean_ by the mediastinum SD.

### Visual image quality assessment for the patient images

The visual image quality was scored by two nuclear radiologists with 2 and 5 years of experience in oncological PET/CT. The two raters reviewed PET/CT images in axial, fusion, and rotation maximum intensity projection (MIP) views. They were blinded to the image reconstruction settings. To reduce the memory effect, the cases were read in a randomized order, and the patient’s identification was removed from the images. The rater scored the images independently without knowing the other rater’s result. If there was a discrepancy in the scores between the two raters, a third rater with 7 years of experience in PET/CT was consulted to reconcile the difference. The SVOSEM group was not included in the visual image quality assessment, because it has 20–25% of the cases with a coefficient of variation (COV) higher than 15% in SVOSEM, suggesting this protocol cannot pass the quality control procedure (Additional file 1: Fig. 2).

The image noise, the lesion depiction, and the overall image quality were scored with a 5-point scale. An image noise score of 1, 2, 3, 4, and 5 was given to the images with unacceptable, acceptable, normal, very good, and excellent image noise performance, respectively. A lesion depiction score of 1–5 was given to the images with non-diagnostic quality, acceptable lesion detectability but may be equivocal on small lesions, average lesion depiction ability with moderate confidence, good small lesion detection ability, and excellent small lesion detectability with strong confidence to delineate small and low contrast lesions and surrounding structures. An overall image quality score of 1–5 was given to the images with non-diagnostic, acceptable, average, good, and excellent image quality for small lesion depiction and quantification.

### Statistical analysis

The data were presented as mean ± SD. The quantitative data were compared using a two-tailed paired t-test if the data followed the normal distribution according to Shapiro–Wilk normality test. The quantitative data with non-normal distribution and visual image quality scores were compared using Wilcoxon signed-rank test. The *p* value was adjusted with Benjamini & Hochberg procedure to reduce the chance of type I error due to the multiple pairwise comparisons between the reconstruction groups. The inter-rater agreements of the image quality scores were tested by Cohen’s Kappa test. The scores unified by the third rater were used in the comparison of the reconstruction groups. A *p* value of 0.05 was deemed as statistically significant. All data were processed with Microsoft Excel version 2016 and R statistical package version 4.0.5. The line profiles of the lesions were extracted using AMIDE Medical Image Data Examiner version 1.0.4 and plotted with Microsoft Excel version 2016.

## Results

### Results of the phantom study

**Fig. 1:**
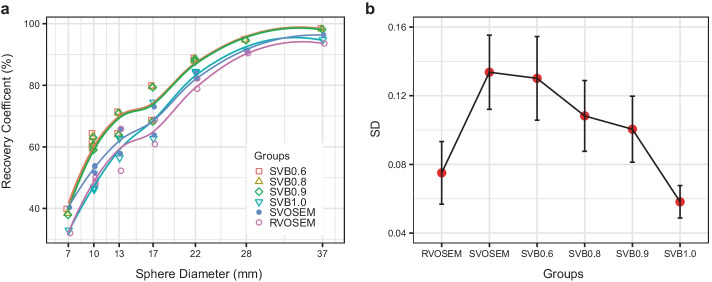
Recovery coefficients (RC) and the standard deviation (SD) of the CTN phantom. **a** The RCs were plotted against the diameter of the spheres. The trend lines were fitted with local polynomial regression model for each group. The hot spheres in the phantom background had higher RCs than those in the polystyrene-filled chambers with the same diameter. The difference of the RC is most obvious at the diameters of 10, 13, 17, and 22 mm. The trend lines pass through in the middle of the two data points of the RCs. **b** The mean of the SD was calculated by averaging the SDs from eight VOIs placed in the phantom background and plotted for each reconstruction group (red dot). The error bar showed the standard deviation of the SD that was also calculated from eight VOIs placed in the phantom background. A narrower error bar represents that the image noise was more homogeneous across different regions in the phantom background

The RC of the hot spheres and the SD of the VOIs in the phantom study are plotted in Fig. 1. All RCs increased with the increase in the diameter of the sphere. However, SVB0.6, SVB0.8, and SVB0.9 groups had higher RCs than the SVOSEM group. And the SVOSEM group had a higher RC than the RVOSEM group. Therefore, BPL reconstructions further improved the RC in addition to the gain from small voxel reconstructions. The SD was not statistically different between SVB0.6 and SVOSEM group (*p* = 0.72). It was smaller in SVB0.8, SVB0.9, and SVB1.0 groups compared to the SVOSEM group (all *p* < 0.05), which showed the noise reduction capability of SVB reconstruction. The SD of RVOSEM was lower than SVB0.6, SVB0.8, and SVB0.9 (all *p* < 0.05), but not statistically different from the SVB1.0 group (*p* = 0.1). The image noise of the phantom is lower than that of the patients (presented in the following sections), especially in the RVOSEM groups. This may be caused by the relatively smaller size of the phantom compared to the patient size.

### Patient characteristics

A total of 24 lung lesions with a diameter of 1.1 ± 0.3 cm (range 0.5–1.8 cm) or a volume of 0.5 ± 0.4 cm^3^ (range 0.06–1.5 cm^3^) measured with CT were included in the study. The average patient weight was 65.8 ± 12.4 kg (range 43–85 kg). Injected ^18^F-FDG dose was 341.3 ± 68.6 MBq (range 262.1–617.1 MBq). The original tumor sites and other clinically relevant information are found in Table 1. The computation time for a typical whole body SVB reconstruction was about 10 min using a computer with an Intel CPU (Xeon E5-2620 V4 @ 2.10 GHz) and two graphics cards (NVIDIA Quadro RTX5000).

**Table 1:** Patient characteristics

Characteristics	Value
Age	65.0 ± 11.7 years
Sex	7 women and 17 men
Height	1.67 ± 0.07 m
Weight	65.8 ± 12.4 kg
Injected activity	341.3 ± 68.6 MBq
Uptake time	60.2 ± 12.3 min
*Original sites*	
Adrenal gland	1
Breast	1
Cervix	1
Colorectum	3
Esophagus	3
Kidney	1
Liver	3
Lung	9
Prostate	1
Stomach	1

*Data are presented as mean ± SD

^#^Data are presented as the patient counts

### Results of quantitative image evaluation for the patient study

**Fig. 2:**
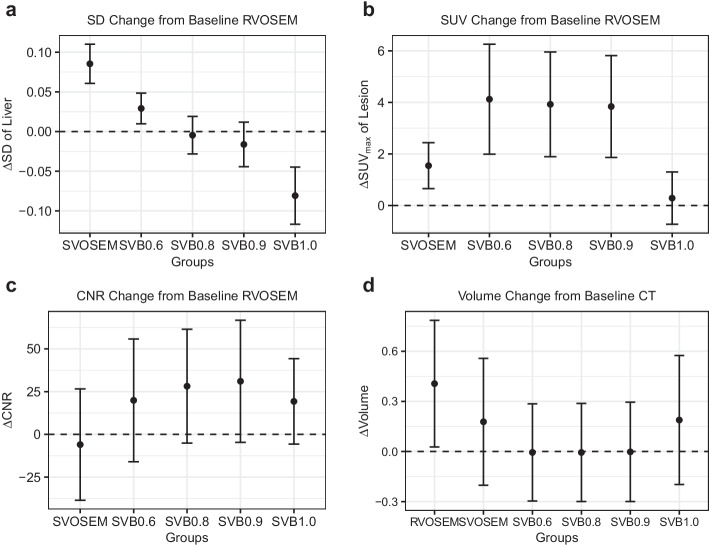
Change of SD, SUV_max_, and CNR in SVB and SVOSEM groups from the baseline (the RVOSEM group) and PET-derived volume change from the baseline (CT measurement) in the patient study. The difference was calculated by subtracting the value of the baseline group from each reconstruction group for every patient. The mean (dot) and standard deviation (error bar) of the difference were calculated over all patients or lesions for each metric and plotted in the figure. The baseline group was RVOSEM for calculating the difference of SD, SUV_max_ and CNR. The baseline group was CT measurement in the calculation of the difference of the lesion volume

Table 2 shows the results of SUV_max_, SUV_mean_, SD, TBR, CNR, and tumor volume measurements. RVOSEM, SVOSEM, and all SVB groups had similar SUV_mean_ in the liver and the mediastinum (all *p* > 0.24 and > 0.14). SVB0.6 had higher SDs in the liver and the mediastinum than RVOSEM, but much lower than SVOSEM. The SDs decreased along with the increase in the penalty and became equivalent to RVOSEM at SVB0.8 and SVB0.9 in the liver (Fig. 2a). The SD of SVB1.0 was lower than RVOSEM. Additional file 1: Tables 2 and 3 provide the *p* values from the paired comparison of the SDs between reconstruction groups.

**Table 2:** Quantitative results of the patient study

	RVOSEM	SVOSEM	SVB0.6	SVB0.8	SVB0.9	SVB1.0	CT
SUV_mean_ of Liver	2.2 ± 0.3	2.2 ± 0.3	2.2 ± 0.3	2.2 ± 0.3	2.2 ± 0.3	2.2 ± 0.3	
SD of Liver	0.20 ± 0.05	0.28 ± 0.07	0.22 ± 0.05	0.19 ± 0.04	0.18 ± 0.04	0.11 ± 0.03	
SUV_mean_ of Mediastinum	1.6 ± 0.3	1.6 ± 0.3	1.6 ± 0.3	1.6 ± 0.3	1.6 ± 0.3	1.6 ± 0.3	
SD of Mediastinum	0.12 ± 0.05	0.19 ± 0.08	0.15 ± 0.05	0.12 ± 0.05	0.12 ± 0.06	0.07 ± 0.03	
SUV_max_ of Lesion	4.6 ± 3.4	6.1 ± 3.3	8.7 ± 3.9	8.5 ± 3.9	8.4 ± 3.9	4.9 ± 4.0	
CNR of Lesion	32.5 ± 44.9	26.5 ± 18.8	52.4 ± 30.6	60.7 ± 34.9	63.5 ± 38.1	51.8 ± 52.4	
TBR of Lesion	2.1 ± 1.6	2.8 ± 1.5	4.0 ± 1.8	3.9 ± 1.8	3.8 ± 1.8	2.3 ± 1.8	
Volume of Lesion (cm^3^)	0.9 ± 0.4	0.7 ± 0.4	0.5 ± 0.4	0.5 ± 0.4	0.5 ± 0.4	0.7 ± 0.4	0.5 ± 0.4

^*^Data are presented as mean ± SD

The SUV_max_ of the lesion was greater in SVB0.6, SVB0.8, and SVB0.9 compared to RVOSEM and SVOSEM. However, no difference was found between SVB1.0 and RVOSEM. The SUV_max_ of SVOSEM was higher than that of RVOSEM, but it was lower than that in SVB0.6, SVB0.8 and SVB0.9 (Fig. 2b). See Additional file 1: Section III for the *p* value in the comparisons of SUV_max_ between groups. In summary, SVB0.8 and SVB0.9 outperformed SVOSEM with higher contrast (SUV_max_) and lower image noise (SD).

SVB0.6, SVB0.8, and SVB0.9 groups had higher TBRs than RVOSEM. SVB1.0 and RVOSEM had comparable TBRs (*p* = 0.3, Additional file 1: Table 5). The mean CNRs of all SVB groups were higher than that of RVOSEM (Fig. 2c). The SVOSEM group achieved higher TBR than the RVOSEM group. However, because the image noise of SVOSEM was high, the mean CNR was slightly smaller than that of RVOSEM (Fig. 2c), although it was not statistically significant (*p* = 0.47, Additional file 1: Table 6).

The MTV of the lesion was smaller in all SVB groups compared to the RVOSEM group (all *p* < 0.001). The MTVs derived from SVB0.6, SVB0.8, and SVB0.9 groups were statistically equivalent to that measured on CT images (all *p* > 0.24). However, the volumes derived from RVOSEM, SVOSEM, and SVB1.0 group were bigger than CT measurements (all *p* < 0.05). Using CT volumetric measurement as the standard reference, the volumes derived from SVB0.6, SVB0.8, and SVB0.9 were more accurate than those from RVOSEM, SVOSEM, and SVB1.0 (Fig. 2d).

### Results of visual image scores for the patient study

**Fig. 3:**
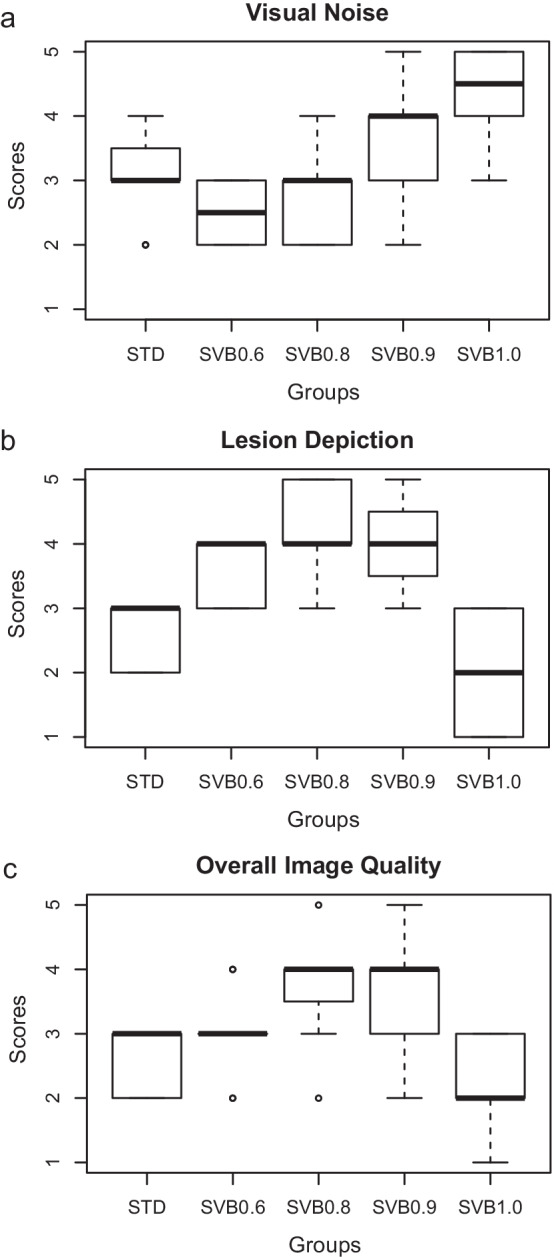
Visual image scores. **a** Image noise; **b** lesion depiction; **c** overall image quality

**Fig. 4:**
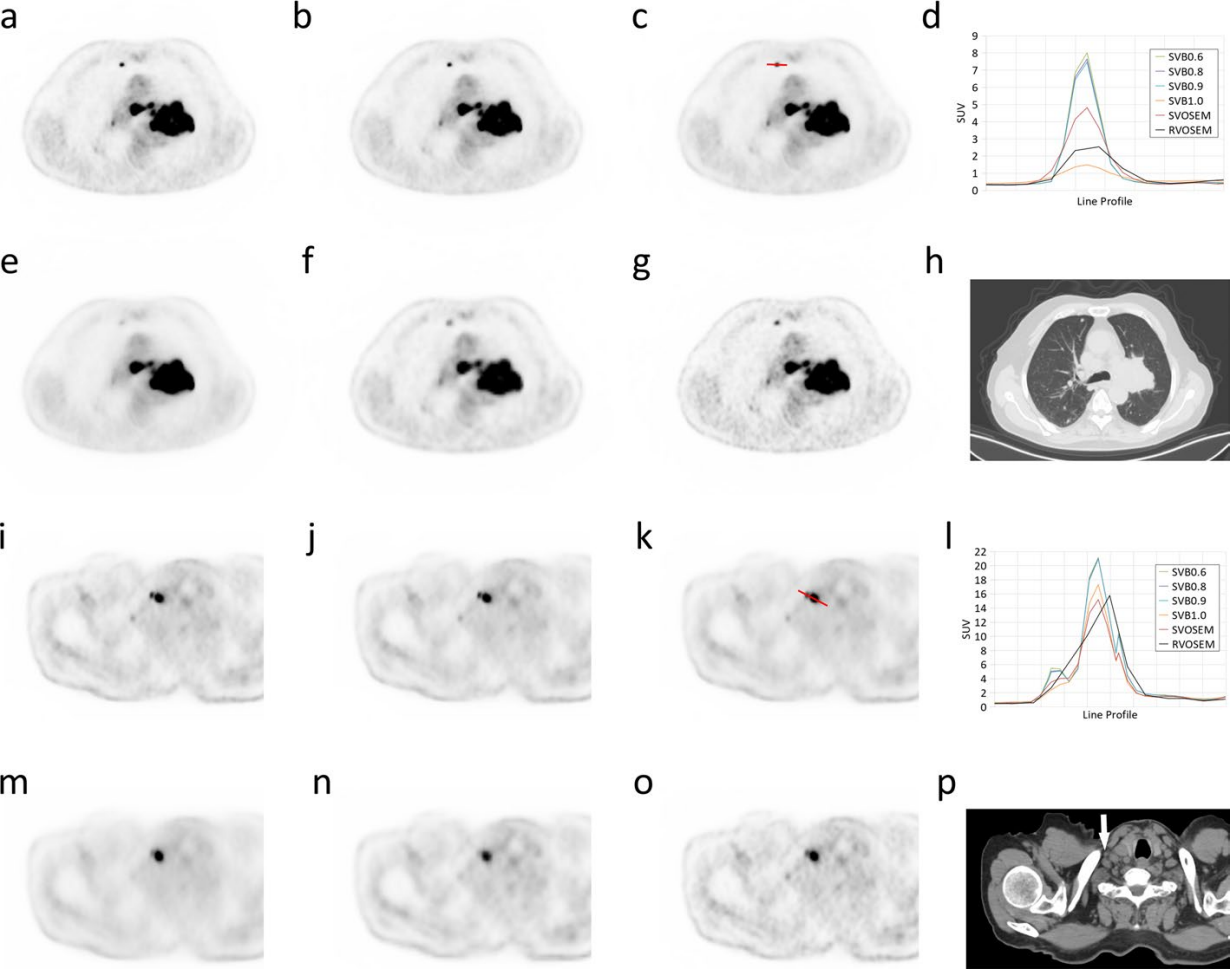
An 83-year man with lung cancer. The images of a lung nodule in the middle of the right lung next to the sternum were shown for SVB0.6 (**a**), SVB0.8 (**b**), SVB0.9 (**c**), SVB1.0 (**e**), RVOSEM (**f**), SVOSEM (**g**), and CT (**h**). The nodule has a diameter of 0.56 cm measured on CT axial view. The line profiles of the lung nodule were plotted on panel d for each reconstruction group. A red line was placed in panel c to illustrate the location where the line profile was generated. The lesion contrast was improved, and the image noise was similar in SVB0.8 and SVB0.9 groups compared to RVOSEM. Two ^18^F-FDG-avid infraclavicular lymph nodes were identified on the same patient. The PET images were shown for SVB0.6 (**i**), SVB0.8 (**j**), SVB0.9 (**k**), and SVOSEM (**o**). The CT image reveals two round-shaped soft tissue intensity nodes in the panel p. However, the node pointed by a white arrow was less appreciable on the images of SVB1.0 (**m**) and RVOSEM (**n**). The fusion views of PET/CT for the lymph nodes were provided in Additional file 1: Fig. 3 for SVB reconstructions. The line profiles of the lymph nodes for each reconstruction group were shown in panel l. The location of the line profile is illustrated in (**k**) (red line)

The visual noise, lesion depiction, and overall image quality scores are shown in Fig. 3. The visual noise score was improved with a higher penalty factor (Fig. 3a). The visual noise score of SVB0.6 was inferior to that of RVOSEM (*p* < 0.001), and the visual noise score of SVB0.8 was tied with RVOSEM (*p* > 0.11). SVB0.9 and SVB1.0 had superior visual noise scores (*p* < 0.01 and < 0.001). SVB0.6 and SVB0.8 had better lesion depiction scores than RVOSEM (both *p* < 0.001). Further increasing the penalty factor results in a decrease in lesion depiction scores due to the over-smoothing phenomenon suggested by the raters (Fig. 4). Although SVB0.9 was still better than RVOSEM, SVB1.0 had an inferior score of the lesion depiction (*p* < 0.01). The overall image quality score of SVB0.8 and SVB0.9 was superior to RVOSEM (both *p* < 0.001). It was worth noting that the overall image quality score of RVOSEM was slightly lower than that of SVB0.6 (*p* = 0.044), which suggests SVB0.6 might be acceptable by the radiologists. The inter-rater agreement was substantial for visual noise, lesion depiction, and overall image quality scores (κ = 0.695, 0.697, and 0.771, respectively).

## Discussion

The small voxel reconstruction with the BPL algorithm improved the contrast recovery, volumetric measurement accuracy, the lesion depiction capability of small lesions with a diameter of ≤ 2 cm. Our phantom study showed the recovery coefficient was more accurate with SVB reconstructions compared to the RVOSEM reconstruction. Our patient study showed that SVB reconstructions delivered superior contrast, CNR, and TBR that could improve the small lesion detection. Furthermore, the MTVs derived from SVB reconstructions were more accurate using CT measurements as the standard reference while RVOSEM reconstruction over-estimated the volume. A penalty factor between 0.8 and 0.9 can deliver the optimal image quality that had lower image noise, improved quantification accuracy, and superior image quality scores compared to RVOSEM. In summary, SVB reconstruction with a penalty factor of 0.8–0.9 can deliver high-quality images that may benefit the small lesion detection and increase quantification accuracy.

The value of small voxel reconstruction is its ability to delineate fine structures and hence improve the detection of small lesions. Consistent with the previous studies [5, 18, 19], our phantom study showed the hot spheres achieved higher contrast recovery with SVOSEM and SVB reconstructions compared to RVOSEM. This result was further confirmed in our patient study. Moreover, our data support that the small voxel reconstruction provides finer lesion delineation that may not be seen in RVOSEM (Fig. 4). Therefore, the results of the present study and previous studies support that the small voxel reconstruction improved the small lesion detection through high lesion contrast or TBR. Furthermore, our results showed that small voxel reconstructions resulted in smaller MTVs than RVOSEM, which was also found in another study of small voxel reconstruction [7]. Moreover, using CT-derived volume as the standard reference, our results showed SVB reconstructions provide accurate MTV measurement while RVOSEM over-estimates small lesion MTV. Therefore, SVB reconstruction can provide high accuracy for the small lesion quantification, which explains the improvement in the test–retest reliability [20], FDG uptake evaluation in fine structures [21], and tumor characterization with radiomics [22].

The BPL reconstruction can improve the lesion detectability and the quantification accuracy for small pulmonary nodules [11–13, 23], metastases [24–26], and other tumors [27]. The improvement in the small lesion detection depended on the penalization factor, tumor size, contrast, and CNR [14, 15]. Consistent with the previous studies [11–13, 24–26], our study found that the detectability and the quantification accuracy of small lung lesions were improved in SVB reconstructions. In contrast, our study applied BPL with a larger penalty to accommodate the image noise of small voxel reconstruction. We used 2 × 2 × 2 mm^3^ voxels that received only 1/8 of counts of 4 × 4 × 4 mm^3^ voxels, which led to a significant increase in image noise, as shown in the comparison between SVOSEM and RVOSEM. However, BPL successfully reduced the image noise in the SVB groups to the level of the RVOSEM group with a much larger penalty factor than the previous study reported [9]. The need for a larger penalty was also suggested in other BPL studies under the condition of low count statistics or acquisition time dose product [28, 29]. Nevertheless, our results showed that SVB reconstruction with a penalty factor of 0.8–0.9 has a net improvement in the small lesion contrast and CNR and visual score compared to SVOSEM. Therefore, our data support the combination of improved contrast recovery accuracy of SVB reconstruction and the power of BPL in the noise reduction contributes to this net improvement.

Trägårdh et al. [29] evaluated the influence of the penalty factor of block sequential regularized expectation maximization (BSREM) and the product of injected activity and acquisition time (IAAT) on the small lesion detection using ^18^F-FDG PET/CT. They found that a high small lesion detection rate and image quality could be obtained with BSREM reconstructions and a penalty factor of 500–600 while the IAAT could be reduced to 6. In the present study, we found a penalty factor of 0.8–0.9 could achieve the optimal image quality for small lesion detection when IAAT was set at 10. The two studies used two BPL algorithms and two voxel sizes: BSREM vs. TVREM and 2.7 × 2.7 × 2.8 vs. 2 × 2 × 2 mm^3^, which may cause the difference of the penalty factors and IAAT. Yang et al. [9] showed TVREM reconstruction with a penalty factor of 0.14–0.21 provided the optimal image quality for detecting the lesion of prostate cancer using ^68^ Ga-PSMA PET and a reconstructed voxel size of 3.1 × 3.1 × 3 mm^3^. Our study also used TVREM reconstruction, but on small lung tumors using ^18^F-FDG PET with a smaller voxel size of 2 × 2 × 2 mm^3^. Different tracers and voxel sizes may explain the reason a larger penalty factor is recommended in the present study. Further studies are needed to investigate the optimal voxel size and the penalty factors for the tracers beyond ^18^F-FDG.

The objective of our study was to push the boundary of small lesion detection with a reconstruction scheme optimized for spatial resolution. Therefore, we selected a small voxel size of 2 × 2 × 2 mm^3^ in the SVB groups. Although this selection might improve spatial resolution, the SVOSEM group had much higher image noise than the RVOSEM group. Although the lesion contrast was improved, the CNR of SVOSEM was not superior to RVOSEM. Moreover, the excessive image noise of SVOSEM might lead to 20–25% of the examinations that exceed the acceptance level of the quality control criteria, i.e., 15% COV in the liver (Additional file 1: Fig. 2). Therefore, BPL was applied on small voxel reconstruction to reduce the noise by necessity. Due to the satisfactory image quality performance, RVOSEM with 4 × 4 × 4 mm^3^ voxels was used as the standard protocol in routine practice and therefore suitable to serve as the baseline in the comparison with SVB reconstructions. In contrast with the previous studies [11–13, 24] that only compared BPL and OSEM at the same voxel size, our study involved two voxel sizes. Therefore, we can show that SVB reconstruction improves not only lesion CNR but also spatial resolution, which had the potential to detect more small lesions (Fig. 4i–p).

SVB reconstruction may have an impact on the image interpretation criteria and therapy evaluation as previous small voxel and BPL studies suggested [18, 30, 31]. Our results showed SVB reconstruction improved the tumor quantification accuracy and potentially the test–retest reliability, which would benefit the therapy evaluation in follow-up studies. On the other hand, increased lesion SUV with advanced reconstruction techniques may lead to more lesions being classified as malignancy or upgrade the stage of the diseases using the threshold-based interpretation criteria, which raised concerns [32, 33]. To address these issues, Teoh EJ et al. [11] suggested a higher SUV threshold in the interpretation. In another study, Wu Z et al. [23] proposed a formula to calibrate SUV based on lesion size. Our results of the phantom and the patient study showed increasing the penalty to SVB1.0 could make the CR and lesion SUV comparable to that of the RVOSEM reconstruction group, which could be an alternative solution to address the concern on the elevated SUV. Although it has not been tested yet, the reconstruction protocol of RVOSEM can be fit in the harmonization protocols [34]. Therefore, it is also possible to cooperate SVB reconstruction with a high penalty into the harmonization procedure. As our results showed, the images of SVB1.0 had lower image noise than RVOSEM and therefore, resulting in higher CNR of the lesion. It may provide additional benefit to the radiologists in daily practice on the reading efficiency if a harmonized SVB protocol is established. However, more studies are needed to explore the potentials of the harmonization of SVB reconstruction that allows SVB reconstruction to generate consistent results in multi-center trials.

Our study has several limitations. Our patient study included only the lesions in the lungs. The CTN phantom has seven hot spheres in the phantom background to simulate the lesions in the mediastinum, heart, and other non-lung tissues in the chest (Additional file 1: Fig. 1). Our data showed the CRs of the spheres in the phantom background were higher than those in the lung background using the same SVB reconstruction setting (Fig. 1). It indicates that SVB reconstruction improves the lesion contrast in a warm background and, therefore, has the potential to be used in the other body regions. However, further patient studies are needed to validate it in the other body regions. The advance of choosing lung lesions was that a clean segmentation could be obtained on non-contrast CT. The volume derived from CT segmentation can be served as the reference for MTV whose “true” value is often not available in the other organs. On the other hand, the respiratory motion could lead to volume overestimation. However, the MTVs of SVB0.8 and SVB0.9 groups were smaller than that of the RVOSEM group using the same segmentation threshold, which suggests SVB0.8 and SVB0.9 derived MTVs are more accurate for the small lesions. Our study cohort is small. A sub-group analysis was not performed to investigate the optimal penalty factors for different lesion volumes. Furthermore, we only investigated one voxel size—2 mm. Several studies [5, 7, 14, 19] had applied a voxel size between 1 and 2 mm that were all below the current procedure guideline recommendation of 3–4 mm [1]. The voxel size can be further optimized for the small lesion detection and quantification in future studies. An open question remains: how to harmonize the emerging reconstruction schemes with the consideration of the difference of PET system sensitivity and theoretic spatial limit of a realistic PET scanner.

## Conclusions

Our study demonstrated that combining small voxel and BPL reconstruction improved the small lesion contrast, image noise performance, contrast recovery, and volumetric quantification accuracy. SVB reconstruction with a penalization factor of 0.8–0.9 provided the optimal contrast-to-noise ratio, hence the optimal small lesion detectability.

## Supplementary Information

**Additional file 1**. **Supplementary figure 1.** Illustration of CTN chest phantom and the placement of the volume of interest (VOI). The spheres with various colors indicate the locations of VOI for measuring the hot spheres (a and c) and background noise (b and d) on top of the virtual rendering of the CT images (a and b) and maximum intensity projection (MIP) view of the PET images (c and d). **Supplementary table 1.** P-value of pairwise comparisons for SDs between the reconstruction groups using paired t test. **Supplementary figure 2.** The boxplot of coefficient of variation (COV) for the liver and the mediastinum in the patient study. The mean and the standard deviation (SD) of SUV were measured from a 3-cm- and a 1.5-cm-diameter sphere region of interest (ROI) on a homogeneous area in the liver and mediastinum, respectively. The COV was calculated by dividing the SD by the mean of SUV values in the ROI. The three horizontal dot lines marked 10% and 15% COV levels. According to the 18F-FDG PET/CT protocol of Uniform Protocols for Imaging in Clinical Trials (UPICT), 10% and 15% were the recommendation of ideal and acceptance level for quality control. The routine-voxel OSEM (RVOSEM) and small-voxel BPL (SVB) groups with a penalty factor of 0.8 - 1.0 were all within 15% COV limit and had a majority proportion (more than 75% percentile) of the cases with a COV of <10%. The small-voxel OSEM (SVOSEM) group had 5 out of 24 (20.8%) cases with a COV of >15% in the liver and 6 out of 24 (25%) cases with a COV of >15% in the mediastinum. **Supplementary table 2.** P-value of paired t test in the pairwise comparisons between the reconstruction groups for the standard deviation (SD) of pixel values in the ROI in the liver. **Supplementary table 3.** P-value of paired t test in the pairwise comparison between the reconstruction groups of the standard deviation (SD) in the mediastinum. **Supplementary table 4.** P-value in the comparison of SUV_max_ of the lesions using pairwise Wilcoxon signed-rank test between the reconstruction groups. **Supplementary table 5.** P-value of pairwise comparisons of TBR between the reconstruction groups using Wilcoxon signed-rank test. **Supplementary table 6.** P-value of pairwise comparisons for CNR using Wilcoxon signed-rank test. **Supplementary table 7.** P-value of pairwise comparisons for lesion volume using Wilcoxon Signed-Rank Test.

## Data Availability

The datasets generated during and/or analyzed during the current study are not publicly available but are available from the corresponding author on reasonable request.
