# Supplementary material for: Small lesion depiction and quantification accuracy of oncological 18F-FDG PET/CT with small voxel and Bayesian penalized likelihood reconstruction
Source: EJNMMI Phys. 2022 Mar 26;9:23. doi: 10.1186/s40658-022-00451-5 (PMC8964871; doi:10.1186/s40658-022-00451-5)
Supplement: Supplementary file 1 — Additional file 1. Supplementary figure 1. Illustration of CTN chest phantom and the placement of the volume of interest (VOI). The spheres with various colors indicate the locations of VOI for measuring the hot spheres (a and c) and background noise (b and d) on top of the virtual rendering of the CT images (a and b) and maximum intensity projection (MIP) view of the PET images (c and d). Supplementary table 1. P-value of pairwise comparisons for SDs between the reconstruction groups using paired t test. Supplementary figure 2. The boxplot of coefficient of variation (COV) for the liver and the mediastinum in the patient study. The mean and the standard deviation (SD) of SUV were measured from a 3-cm- and a 1.5-cm-diameter sphere region of interest (ROI) on a homogeneous area in the liver and mediastinum, respectively. The COV was calculated by dividing the SD by the mean of SUV values in the ROI. The three horizontal dot lines marked 10% and 15% COV levels. According to the 18F-FDG PET/CT protocol of Uniform Protocols for Imaging in Clinical Trials (UPICT), 10% and 15% were the recommendation of ideal and acceptance level for quality control. The routine-voxel OSEM (RVOSEM) and small-voxel BPL (SVB) groups with a penalty factor of 0.8 - 1.0 were all within 15% COV limit and had a majority proportion (more than 75% percentile) of the cases with a COV of <10%. The small-voxel OSEM (SVOSEM) group had 5 out of 24 (20.8%) cases with a COV of >15% in the liver and 6 out of 24 (25%) cases with a COV of >15% in the mediastinum. Supplementary table 2. P-value of paired t test in the pairwise comparisons between the reconstruction groups for the standard deviation (SD) of pixel values in the ROI in the liver. Supplementary table 3. P-value of paired t test in the pairwise comparison between the reconstruction groups of the standard deviation (SD) in the mediastinum. Supplementary table 4. P-value in the comparison of SUVmax of the lesions using pairwise Wilcoxon signed-rank [file 40658_2022_451_MOESM1_ESM.pdf]

Supplementary to Small Lesion Depiction and Quantification Accuracy of oncological 18F-FDG PET/CT with Small Voxel and Bayesian Penalized Likelihood Reconstruction

Section I: Total Variation Regularized Expectation Maximization

In Total Variation Regularized Expectation Maximization (TVREM), the penalized likelihood function is written as follows:

$$\hat{f} = \operatorname{argmax}_{f\geq 0}\left[\sum_{ij} - p_{ij}f_j + \sum_i c_i \ln\left(\sum_j p_{ij}f_j\right) - \sum_j \gamma_j \cdot U(f_j)\right] \quad (1)$$

$$\gamma_j = g(NEC, sns_j) \cdot\beta \quad (2)$$

$$U(f) = \sum_{x,y,z} |\nabla f| \quad (3)$$

where i and j are the indexes of the projection bins and the image pixels, respectively. f is the estimated image. c_i are the measured emission data. p_{ij} is the system matrix indicating the counts emitted from the jth image pixel detected by the ith projection bin. γ_j is a parameter of regularized strength. U is the total variation penalization of the pixels in the neighborhood. g is a function of noise equivalent counts (NEC) and sns_j, it can be described as

$$g_j \propto NEC^a \cdot sns_j^b \quad (4)$$

where the constants a and b were determined through phantom experiments. The term of NEC evaluates the quality of the data collected under varied activity and acquisition time. sns_j is the spatially varied sensitivity profile. It estimates the noise attributed to the spatially variant sensitivity as well as the attenuation effect. It is voxel dependent. More weight is given to the voxel with lower sensitivity and higher attenuation. Previous studies demonstrated that incorporating the sensitivity map and NEC into the hyper-parameter was an effective way to improve the regularized reconstruction's robustness against noise related to different count levels and spatially variant sensitivity [1].

β is a factor representing penalty strength which is the only parameter that the users could adjust according to their preference on image smoothness. Larger β results in smoother image. Currently, the up limit of the user input value is 1.0. The user input in the user-interface will be scaled by a bilinear transform to generate a real β used in the image reconstruction, that is,

$$\beta = \begin{cases} k_1 \cdot \beta_{input} & \beta_{input} \in (0,0.9] \\ 0.9 \cdot k_1 + k_2 \cdot (\beta_{input} - 0.9) & \beta_{input} \in (0.9,1.0] \end{cases} \quad (5)$$

where k_1 and k_2 are the scaling coefficients.

[1] Xie, H., Y. Lv, and Y. Dong, Impact of sensitivity map and noise equivalent counts on

hyper-parameter selection for regularized image reconstruction. Journal of Nuclear Medicine, 2019. 60(supplement 1): p. 454-454.

Section II: Phantom

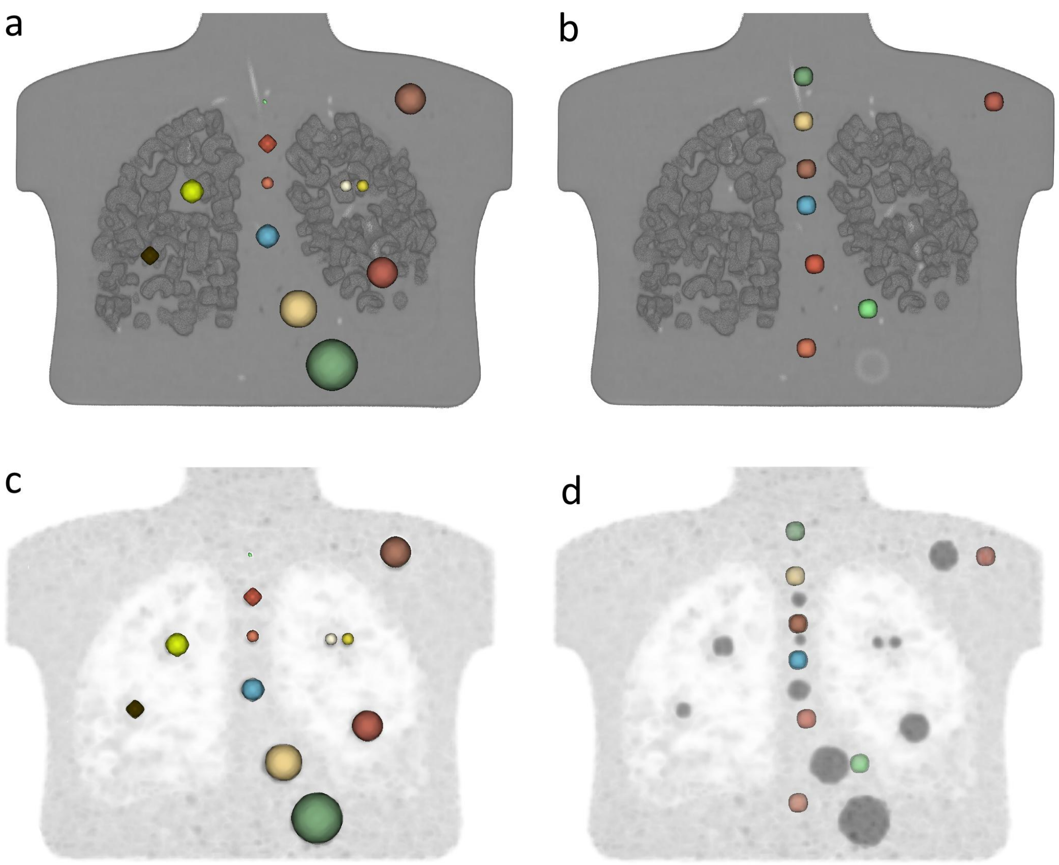

Supplementary figure 1. Illustration of CTN chest phantom and the placement of the volume of interest (VOI). The spheres with various colors indicate the locations of VOI for measuring the hot spheres (a and c) and background noise (b and d) on top of the virtual rendering of the CT images (a and b) and maximum intensity projection (MIP) view of the PET images (c and d).

Supplementary table 1. P-value of pairwise comparisons for SDs between the reconstruction groups using paired t test

	RVOSEM	SVB0.6	SVB0.8	SVB0.9	SVB1.0
SVB0.6	4.7e-06	-	-	-	-
SVB0.8	0.00276	0.03724	-	-	-
SVB0.9	0.01704	0.00672	0.46275	-	-
SVB1.0	0.10437	3.1e-08	2.0e-05	0.00022	-
SVOSEM	1.9e-06	0.71632	0.01704	0.00276	1.9e-08

Section III: Patient Study

a

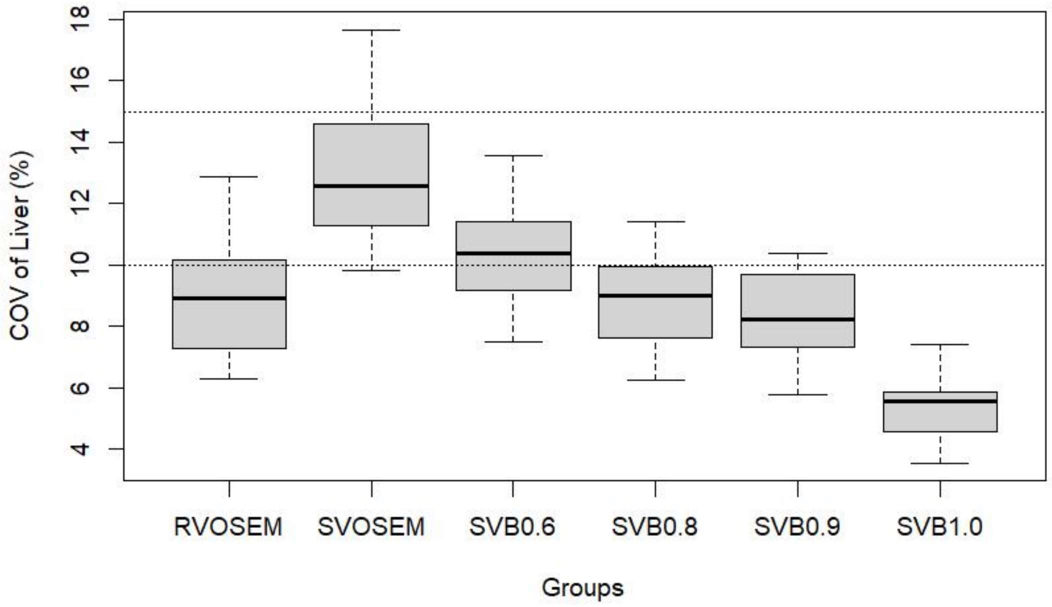

b

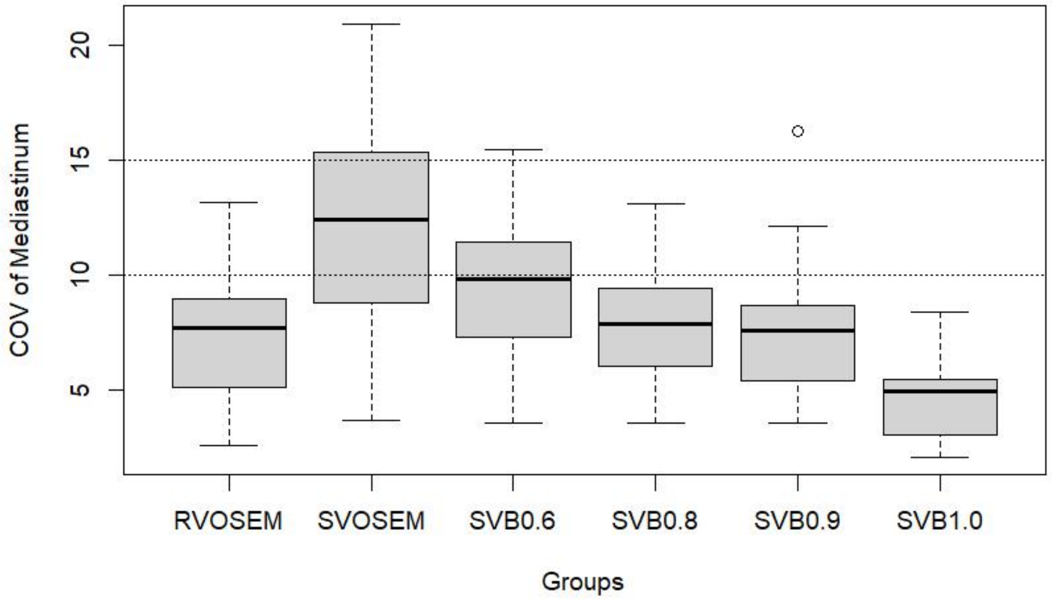

Supplementary figure 2. The boxplot of coefficient of variation (COV) for the liver and the mediastinum in the patient study. The mean and the standard deviation (SD) of SUV were measured from a 3-cm- and a 1.5-cm-diameter sphere region of interest (ROI) on a homogeneous area in the liver and mediastinum, respectively. The COV was calculated by dividing the SD by the

mean of SUV values in the ROI. The three horizontal dot lines marked 10% and 15% COV levels. According to the 18F-FDG PET/CT protocol of Uniform Protocols for Imaging in Clinical Trials (UPICT), 10% and 15% were the recommendation of ideal and acceptance level for quality control. The routine-voxel OSEM (RVOSEM) and small-voxel BPL (SVB) groups with a penalty factor of 0.8 - 1.0 were all within 15% COV limit and had a majority proportion (more than 75% percentile) of the cases with a COV of <10%. The small-voxel OSEM (SVOSEM) group had 5 out of 24 (20.8%) cases with a COV of >15% in the liver and 6 out of 24 (25%) cases with a COV of >15% in the mediastinum.

Supplementary table 2. P-value of paired t test in the pairwise comparisons between the reconstruction groups for the standard deviation (SD) of pixel values in the ROI in the liver

	RVOSEM	SVB0.6	SVB0.8	SVB0.9	SVB1.0
SVB0.6	1.8e-07	-	-	-	-
SVB0.8	0.35	5.1e-13	-	-	-
SVB0.9	0.01	4.9e-12	4.0e-08	-	-
SVB1.0	1.7e-10	1.8e-13	2.4e-13	2.8e-13	-
SVOSEM	1.8e-13	1.7e-11	2.9e-12	5.4e-12	2.4e-13

Supplementary table 3. P-value of paired t test in the pairwise comparison between the reconstruction groups of the standard deviation (SD) in the mediastinum

	RVOSEM	SVB0.6	SVB0.8	SVB0.9	SVB1.0
SVB0.6	0.0015	-	-	-	-
SVB0.8	0.4997	3.0e-08	-	-	-
SVB0.9	0.7299	0.0018	0.7299	-	-
SVB1.0	1.8e-05	2.0e-10	2.0e-10	2.3e-05	-
SVOSEM	3.9e-07	3.7e-07	7.2e-08	1.1e-05	6.2e-10

Supplementary table 4. P-value in the comparison of SUV_{max} of the lesions using pairwise Wilcoxon signed-rank test between the reconstruction groups

	RVOSEM	SVB0.6	SVB0.8	SVB0.9	SVB1.0
SVB0.6	2.0e-07	-	-	-	-
SVB0.8	2.0e-07	4.9e-05	-	-	-
SVB0.9	2.9e-05	3.6e-05	4.7e-05	-	-
SVB1.0	0.33039	2.0e-07	2.0e-07	2.0e-07	-
SVOSEM	2.0e-07	2.0e-07	2.0e-07	2.0e-07	0.00042

Supplementary table 5. P-value of pairwise comparisons of TBR between the reconstruction groups using Wilcoxon signed-rank test

	RVOSEM	SVB0.6	SVB0.8	SVB0.9	SVB1.0
SVB0.6	2.0e-07	-	-	-	-
SVB0.8	2.0e-07	8.2e-06	-	-	-
SVB0.9	2.0e-07	1.6e-06	3.3e-05	-	-
SVB1.0	3e-01	2.0e-07	2.0e-07	2.0e-07	-
SVOSEM	3.6e-07	2.0e-07	2.0e-07	2.0e-07	6e-04

Supplementary table 6. P-value of pairwise comparisons for CNR using Wilcoxon signed-rank test

	RVOSEM	SVB0.6	SVB0.8	SVB0.9	SVB1.0
SVB0.6	0.00300	-	-	-	-
SVB0.8	0.00081	3.9e-05	-	-	-
SVB0.9	0.00051	0.00011	0.00161	-	-
SVB1.0	0.00051	0.47317	0.11665	0.09224	-
SVOSEM	0.47028	8.9e-07	8.9e-07	1.2e-06	0.00384

Supplementary table 7. P-value of pairwise comparisons for lesion volume using Wilcoxon Signed-Rank Test

SVOSEM vs. CT	RVOSEM vs. CT	SVB0.6 vs. CT	SVB0.8 vs. CT	SVB0.9 vs. CT	SVB1.0 vs. CT
0.0279	0.000974	0.236	0.236	0.235	0.030

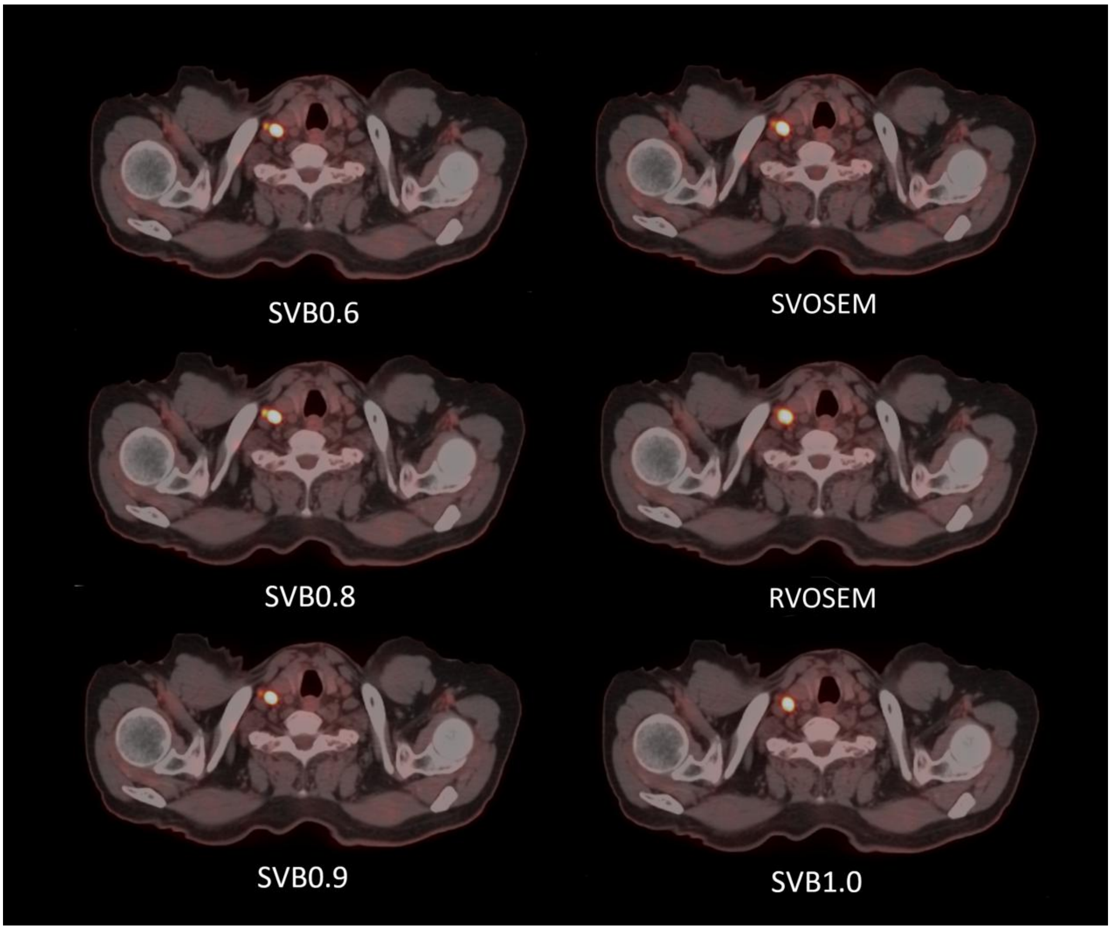

Supplementary figure 3. The fusion views as a supplementary to Figure 4 in the main manuscript. Two ^{18}F-FDG-avid lymph nodes were delineated in SVB0.6, SVB0.8, SVB0.9 and SVOSEM, but not in SVB1.0 and RVOSEM.
